# The role of transient resonances for ultra-fast imaging of single sucrose nanoclusters

**DOI:** 10.1038/s41467-019-13905-9

**Published:** 2020-01-09

**Authors:** Phay J. Ho, Benedikt J. Daurer, Max F. Hantke, Johan Bielecki, Andre Al Haddad, Maximilian Bucher, Gilles Doumy, Ken R. Ferguson, Leonie Flückiger, Tais Gorkhover, Bianca Iwan, Christopher Knight, Stefan Moeller, Timur Osipov, Dipanwita Ray, Stephen H. Southworth, Martin Svenda, Nicusor Timneanu, Anatoli Ulmer, Peter Walter, Janos Hajdu, Linda Young, Filipe R. N. C. Maia, Christoph Bostedt

**Affiliations:** 1 Chemical Sciences and Engineering Division, Argonne National Laboratory, Argonne, IL 60439 USA; 2 Laboratory of Molecular Biophysics, Department of Cell and Molecular Biology, Uppsala University, SE-751 24 Uppsala, Sweden; 3 Chemistry Research Laboratory, Department of Chemistry, Oxford University, 12 Mansfield Rd, Oxford, OX1 3TA UK; 4 European XFEL GmbH, Holzkoppel 4, D-22869 Schenefeld, Germany; 5 Linac Coherent Light Source, SLAC National Accelerator Laboratory, Menlo Park, CA 94025 USA; 6 ARC Centre of Excellence for Advanced Molecular Imaging, La Trobe University, Bundoora, VIC 3086 Australia; 7 Stanford Pulse Institute, SLAC National Accelerator Laboratory, Menlo Park, CA 94025 USA; 8 Computational Science Division, Argonne National Laboratory, Argonne, IL 60439 USA; 9 Department of Physics and Astronomy, Uppsala University, SE-751 20 Uppsala, Sweden; 10 Institut für Optik und Atomare Physik, Technische Universität Berlin, 10623 Berlin, Germany; 11 Department of Physics and James Franck Institute, The University of Chicago, Chicago, IL 60637 USA; 12 Department of Physics and Astronomy, Northwestern University, Evanston, IL USA; 13 Paul-Scherrer Institute, CH-5232 Villigen PSI, Switzerland; 14 LUXS Laboratory for Ultrafast X-ray Sciences, Institute of Chemical Sciences and Engineering, École Polytechnique Fédérale de Lausanne (EPFL), CH-1015 Lausanne, Switzerland

**Keywords:** Imaging techniques, Free-electron lasers, Atomic and molecular interactions with photons

## Abstract

Intense x-ray free-electron laser (XFEL) pulses hold great promise for imaging function in nanoscale and biological systems with atomic resolution. So far, however, the spatial resolution obtained from single shot experiments lags averaging static experiments. Here we report on a combined computational and experimental study about ultrafast diffractive imaging of sucrose clusters which are benchmark organic samples. Our theoretical model matches the experimental data from the water window to the keV x-ray regime. The large-scale dynamic scattering calculations reveal that transient phenomena driven by non-linear x-ray interaction are decisive for ultrafast imaging applications. Our study illuminates the complex interplay of the imaging process with the rapidly changing transient electronic structures in XFEL experiments and shows how computational models allow optimization of the parameters for ultrafast imaging experiments.

## Abstract (web summary)

X-ray free electron lasers provide high photon flux to explore single particle diffraction imaging of biological samples. Here the authors present dynamic electronic structure calculations and benchmark them to single-particle XFEL diffraction data of sucrose clusters to predict optimal single-shot imaging conditions.

## Introduction

X-ray free-electron laser (XFEL) pulses have inspired new approaches for imaging single nanoscale structures^1^, biological samples^2^, aerosols^3^, and inorganic clusters^4^. The combination of the high number of photons (${\approx} 1{0}^{12}$) per pulse with femtosecond pulse lengths allows the recording of diffraction images of nanometer-sized samples with single x-ray flashes. From the two-dimensional x-ray diffraction patterns, three-dimensional information can be obtained^5^ and biological function revealed^6^. Combining the single-shot imaging approach with an optical pump laser yields insight into non-thermal processes on the nanoscale with femtosecond resolution^7^. New approaches based on x-ray Fourier transform holography^8^ promise efficient reconstruction of the real space information from diffraction images of gas phase specimens^9^.

However, single-particle imaging experiments remain challenging^10^. To attain nanometer and potentially even sub-nanometer resolution, high quality diffraction images with signal to large momentum transfers **q** [nm${}^{-1}$] are needed. In linear scattering theory, away from resonances the total scattered intensity scales with wavelength squared (${\lambda }^{2}$)^11^, often leading to the conclusion that single-shot imaging experiment should be performed at the longest wavelength supporting the desired resolution. This thought is particularly interesting for imaging biological samples, for which a resolution of a few nanometers can be sufficient. Here, the so called water-window, referring to the range of photon energies between the carbon and oxygen K-edges, is often considered an ideal choice. In this spectral regime, water exhibits a high x-ray transparency and close to the absorption edge of oxygen high contrast images from biological specimens can be obtained at synchrotron sources^12–14^.

Since the early vision for single-shot imaging experiments with XFELs^15^, radiation induced damage has been an important topic. For atoms interacting with an intense XFEL pulse, sequential absorption can be considered a rule of thumb^16^ that can be strongly enhanced through transient resonances^17–19^. For extended systems, the ionization dynamics are altered through the neighboring atoms^20^ and any nanoscale sample in the x-ray focus is transformed into a highly excited state with a large fraction of delocalized electrons^21^ and with strong electron - nuclear coupling^22^. So far, models for single-shot imaging experiments focus on structural sample damage^23^, follow the frozen nuclei approach^24,25^, include only generalized electron dynamics^26^, or are limited to small systems sizes^27,28^.

**Fig. 1:**
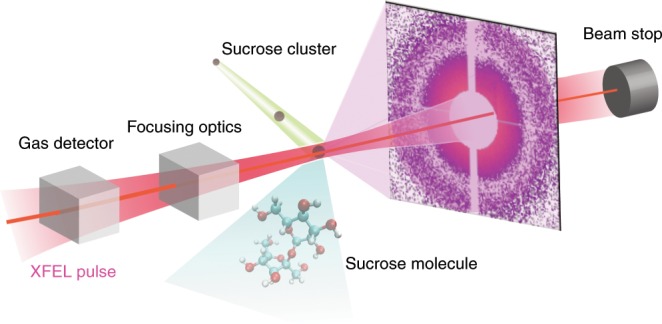
Experimental setup. Experimental setup for the single shot diffractive imaging measurements of sucrose clusters. The clusters intersect the focused FEL pulse and their coherent diffraction image is recorded with a large area pixel detector. Upstream of the focusing optics the energy of each XFEL pulse is measured and recorded with the scattering data. The clusters are small compared to the focus diameter and their location within the focal volume determines the incident fluence on them.

We present full, dynamic scattering calculations of an organic sample, namely a sucrose molecular cluster, with sizes up to 50 nm and compare it to experimental, wavelength-dependent diffractive imaging data, from the water window to the tender x-ray regime. We track the full transient structure of the sample including all active electrons and nuclei with a massive parallel Monte-Carlo/Molecular Dynamics code on the Mira supercomputer^29^. We compare the calculations to single-shot single-particle imaging data obtained at the Linac Coherent Light Source free-electron laser (see Methods) with the setup depicted in Fig. 1 and obtain good agreement. Both the experimental data and theoretical results show that already 1 keV above the oxygen K-edge the scattering response is reduced compared to linear scaling models. At and below the oxygen absorption edge, transient resonances reduce the scattering signal by more than an order of magnitude. Furthermore, the pulse duration is an important parameter for manipulating the scattering response. Our work connects to the start-to-end simulations for single-particle imaging in the hard x-ray regime^30,31^ and dynamic calculations on molecular systems^32^ but we focus on near absorption edge phenomena in the water window and beyond, where transient resonances are important for understanding the scattering response. The water window is loosely defined as the photon energy range from 284 to 540 eV as here water is ~10 times more transparent than organic molecules^12^. We show that for water window imaging experiments employing intense x-ray pulses with pulse duration much longer than the inner-shell lifetimes (e.g. C $\approx$ 10 fs, N $\approx$ 7 fs, O $\approx$ 5 fs), the photon energy needs to be significantly red shifted compared to experiments in the linear regime for optimal imaging.

## Results

### Fluence measurements

**Fig. 2:**
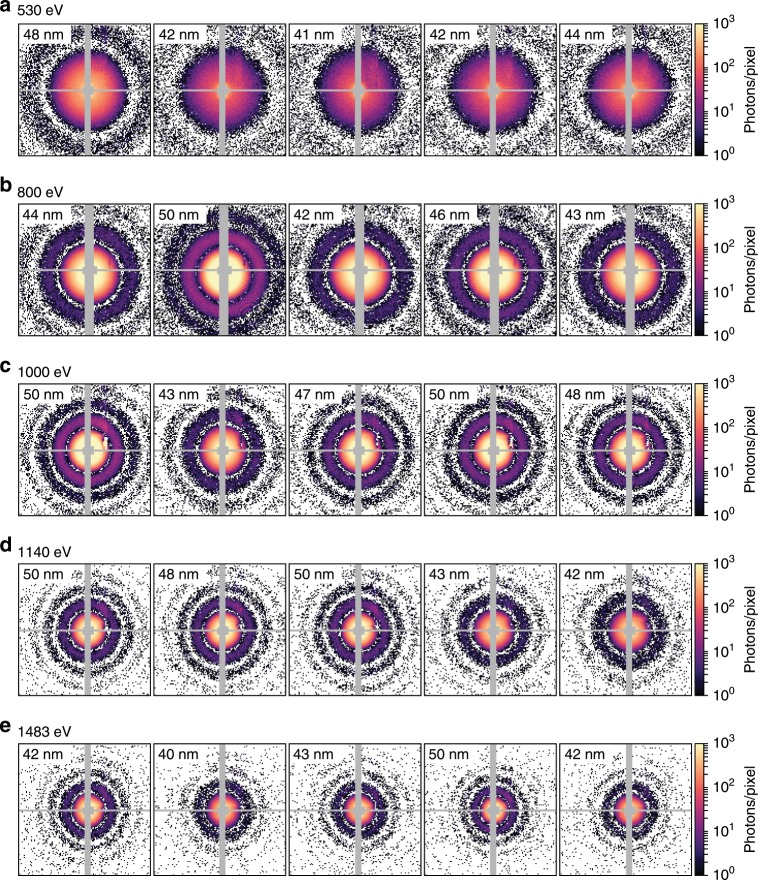
Examples of recorded diffraction images of sucrose clusters. The circular diffraction images indicate spherical particles. With the increasing photon energies 530 eV (**a**), 800 eV (**b**), 1000 eV (**c**), 1140  eV (**d**) and 1483 eV (**e**) additional diffraction fringes become visible. The variations in the fringe locations stem from the particle size distribution.

We recorded single shot diffractive imaging data of sucrose clusters with sizes in the range of 30–70 nm and photon energies ranging from 530 eV in the water window up to 1500  eV. Some of the most intense single-shot coherent images from individual sucrose nanoparticles with sizes around 45 nm for five different photon energies are shown in Fig. 2 (see also Supplementary Note 1). Overall the images are of very high quality with little background scatter and good signal to noise ratio. With increasing photon energy, the number of fringes increases in the images.

For further analysis of the images, we fit the number of scattered photons from single sugar balls within a size range of one standard deviation from the mean cluster size, using the linear scattering theory of a homogeneous sphere (see Methods). An important parameter in this model is the incident fluence ${I}^{o}$ on each particle. This parameter ${I}^{o}$ describes which fluence is required to generate the scattering response of a particle in the absence of light-induced damage.

**Fig. 3:**
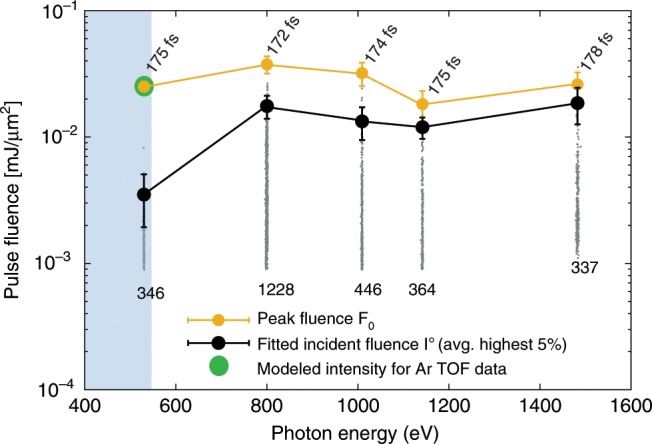
Fluence measurements. Comparison of the beamline peak fluence and the fitted incident fluence from the diffraction images. The fitted incident fluence (${I}^{o}$) is obtained from diffraction images of sucrose clusters with sizes that fall within one standard deviation from the mean. The light gray points are all data and the black markers represent the 5% most intense hits at each photon energy. The peak fluence (${F}_{0}$) is obtained from the beamline data and normalized to the Ar time-of-flight (TOF) data. The error bars of ${I}^{o}$ and ${F}_{0}$ are associated with the maximum uncertainty of the 5% most intense hits (see Supplementary Note 2). The shaded area indicates the water window. The numbers near the yellow points and gray points correspond to the pulse duration and number of recorded diffraction patterns, respectively.

The fitted ${I}^{o}$ data points for the sucrose nanoparticles are plotted as light gray points in Fig. 3. The large variation in ${I}^{o}$ is expected as the incident fluence on each particle depends strongly on the particle position within the x-ray focal volume^21^. The black solid markers in Fig. 3 are the average of the five percent most intense hits, representing a statistically relevant number for the vastly different data set sizes, and they are considered the fitted peak incident fluence of each photon energy point. For comparison the peak fluence from the argon atomic ionization reference measurement (green point) as well as the peak fluence, ${F}_{0}$, determined from the average pulse energies and nominal beamline parameters (yellow markers) are added (see Methods IV A).

The comparison between the peak fluence from the beamline data, ${F}_{0}$, and the fitted incident fluence ${I}^{o}$ in Fig. 3 exhibits a drastic difference at 530 eV, just below the oxygen absorption edge, in addition to an overall decrease in ${I}^{o}$ compared to the beamline data when going from higher to lower photon energies. The large decrease in ${I}^{o}$ indicates a decrease in the number of scattered photons, which can be attributed to a non-linear x-ray response in the sample during the intense x-ray pulse. Earlier work on rare gas model systems showed that under similar conditions the samples are transformed into a highly excited state^20,21^. X-ray pump/x-ray probe data suggests that there is a sizable reduction in scattering intensity already during the x-ray pulse due to the electronic excitations in the target and before the lattice can respond^22^.

The photon energy dependent sucrose imaging data directly shows that the scattering response in intense x-ray pulses is a dynamic process due to the competing absorption and scattering processes. Far above absorption resonances, where the samples become increasingly transparent to the x-ray pulse, the linear model used previously^33–35^ and described in Eq. (1) works satisfactorily. However, near an absorption edge a more detailed description including electron and nuclear changes during the x-ray pulse becomes important.

### Monte-Carlo/Molecular-Dynamics calculations

To understand the scattering response of the sucrose clusters, or more generally organic nanoscale samples, we employed Monte-Carlo/Molecular-Dynamics (MC/MD) calculations^29,36^ on Mira, a BG/Q supercomputer at the Argonne Leadership Computing Facility (see Methods IV C). In our model the interaction of the XFEL pulse with the cluster is treated quantum mechanically, all particles are propagated in time, and the time-dependent form factor including the delocalized electrons of the succrose clusters are calculated. With this approach we can model the full electron and nuclear dynamics in an atomistic manner and calculate the resulting diffraction images in parallel during the full duration of the x-ray pulse under realistic beam parameters. We computationally investigate the scattering response of a sucrose cluster in an intense XFEL pulse as a function of photon energy, pulse duration and fluence. Each sucrose cluster is modeled as a collection of independent atoms while maintaining the stochiometric formula (C${}_{12}$H${}_{22}$O${}_{11}$) and structure of the sucrose molecules. The electronic structure of the three types of atoms are obtained using the Hartree-Fock-Slater model.

We start with computing the scattering response of sugar nanoparticles comprised of 185,193 sucrose molecules in an intense XFEL pulse. The size of these nanoparticles is 50 nm, similar to the experimentally investigated samples. Since each molecule has 182 electrons and 45 nuclei, the calculation for these big clusters requires tracking about 42 million particles.

**Fig. 4:**
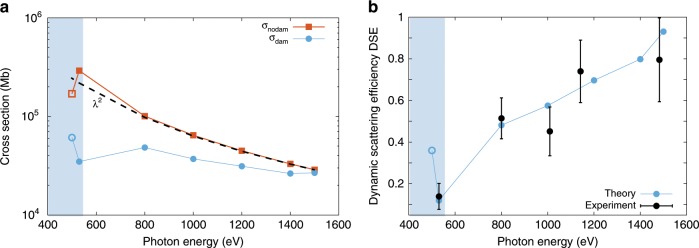
Results from the computational study. **a** Calculated scattering cross sections for a 50-nm sucrose cluster exposed to a 180-fs, 25 $\upmu$J/$\upmu$m${}^{2}$ pulse and comparison of the damaged (blue) to the undamaged (red) clusters as well as classical scaling model (black dashed line). **b** Comparison of dynamic scattering efficiency (DSE) from the calculations (blue) to the experimental results (black markers). The shaded area indicates the water window. The error bars for the experimental results are computed from the uncertainties of ${F}_{0}$ and ${I}^{o}$, as shown in Fig. 3.

Figure 4a shows the calculated scattering cross sections for the particles without any damage, ${\sigma }_{{\rm{nodam}}}$ (red, square markers) as well as the scattering cross section including the full electronic and nuclear dynamics, ${\sigma }_{{\rm{dam}}}$ (blue, round markers). Compared to the experimental data an additional photon energy point at 500 eV deep inside the water window was added. The pulse length was set to 180 fs, the bandwidth to 1%, and the fluence to 25 $\upmu$J/$\upmu$m${}^{2}$, which resembles the modeled peak fluence for the Ar data at 530 eV (see Fig. 3 and Methods).

The calculations for the undamaged particles match the ${\lambda }^{2}$ dependence expected from the linear scattering theory with the exception around the oxygen K-edge and in the water window, where anomalous scattering processes lead to reduced form factors of the oxygen atom, as shown in Fig. 4. In contrast, the calculations including the full electronic and nuclear dynamics exhibit a much flatter cross section over most of the photon energy range, that only partially recovers for the 500 eV data point well below the oxygen edge. From Fig. 4a it becomes immediately clear that the x-ray induced processes have a significant impact on the scattering cross section even as high as 1 keV above an absorption edge that cannot be neglected.

## Discussion

To compare the calculated cross sections with the experimental measurement we employ the Dynamic Scattering Efficiency, DSE, (see Methods) allowing us to directly compare the calculated cross sections with the experimental data. DSE is the ratio of the fitted fluence, ${I}^{o}$, over the peak fluence, ${F}_{0}$, and for the theoretical results DSE is the ratio of the damaged to the undamaged cross sections ${\sigma }_{{\rm{dam}}}/{\sigma }_{{\rm{nodam}}}$. The calculated as well as the experimentally determined Dynamic Scattering Efficiency is shown in Fig. 4b as blue and black filled circles, respectively. Both data sets show a consistent suppression of the scattering in the whole photon energy regime with a pronounced minimum at 530 eV, just below the oxygen K-edge.

The strongest damage is observed in both data sets inside the water window and here even just below the oxygen absorption edge of the sucrose molecule, even though intuitively less absorption and therefore less damage is expected. For reference, the water window is loosely defined to end at the ionization threshold for oxygen in the water molecule around 540 eV^12^. In the sucrose molecule, the lowest resonant energy of the 1s $\to$ ${\pi }^{* }$ transition is in the range of 530–535 eV^37^ and thus within the bandwidth of the 530-eV pulse. In order to investigate this effect further we have added an additional photon energy point of 500 eV in the calculations. Compared to 530 eV, the scattering cross section at 500 eV has greatly recovered (Fig. 4a) and accordingly the damage is reduced again (Fig. 4b). This behavior can be explained with transient resonant phenomena right at the absorption edges.

**Fig. 5:**
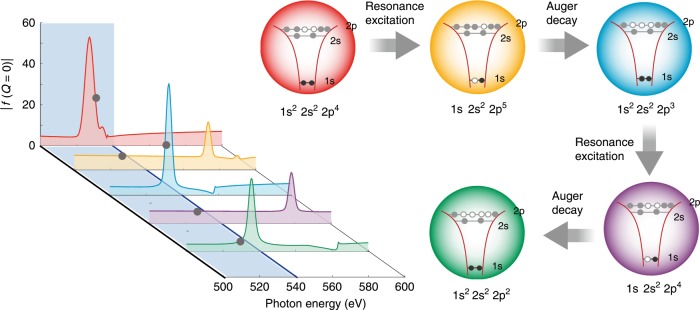
Calculated transient form factors at the resonance. At 530 eV, resonance-mediated pathways induced by a broadband XFEL pulse lead to one or more cascades of core excitation and Auger decay in oxygen atoms. These processes induce rapid changes in the electronic configurations, as well as scattering amplitudes, since the atomic form factor is sensitive to electronic configurations near resonances. The gray dots indicate the absolute values of the complex form factor in the forward direction, $| f(Q=0)|$, at 530 eV. The color of the depicted electronic states corresponds to the color of the calculated form factors.

At 530 eV, even though just below the lowest oxygen absorption feature in the sucrose molecule, resonant mediated pathways can lead to additional ionization events^19,38^. After the initial absorption and Auger decay cycle (∼4-fs lifetime), the pulse can further excite K-shell electrons to the the newly created open 2p subshell vacancies as depicted in Fig. 5. This resonant excitation opens additional ionization channels via one or multiple Auger decay cyclings during the 180-fs x-ray pulse. Employing the Hartree-Fock-Slater model (c.f. Methods and^29^) and assuming a 1% bandwidth of the XFEL pulse, the resonant (1s to 2p) cross section is found to be equal to 1.20 Mbarn whereas the total ionization cross section (from 2s and 2p orbitals combined) is equal to ~0.03 Mbarn, which agrees with the value in ref. ^39^. In other words, the resonant nature of this process increases the absorption cross section 40 times compared to the non-resonant photoionization cross section.

The multiple resonant absorption and Auger decay cycles rapidly increase the number of delocalized electrons in the particle and create highly charged ions with reduced scattering strength (Fig. 5). Energy redistribution^28,40^ and three-body recombination^41^ within the dense nanoplasma leads to high electron kinetic energies and structural damage^7^. At the peak of the pulse the particle has already lost more than 30% of the overall electrons and expanded more than 20% in diameter (see Supplementary Note 6). As a consequence, the overall scattering cross section of the sucrose clusters is suppressed. Because of these x-ray induced ultrafast electron dynamics and structural expansion, the incident pulse effectively scatters off a smaller cluster core and the scattering signals are attenuated (c.f.^7^) which is in agreement with the experimental data.

Besides the photon energy, the x-ray pulse duration is an accessible experimental parameter in XFEL imaging experiments^10^. From the data in Fig. 4 and calculations in Fig. 5 we concluded that the Auger cycling of inner-shell electrons can lead to strong reductions of the scattering signal, underlining the importance of the pulse duration in the current discussion. Further, the inner-shell absorption of intense x-ray pulses is a sequential process and for pulse durations comparable to the inner-shell lifetime, atoms^16^ and clusters^20^ can become transiently x-ray transparent. However, the effect of pulse duration under real imaging conditions has so far mostly been characterized in the hard x-ray regime^30,31,36^, but not in the water window or more generally near absorption edges where resonances play an important role at high x-ray intensities. For experimentalists it can be tempting to choose higher pulse energies over shorter pulse durations. Here we look at the influence of the pulse duration on the scattering response.

For our investigation we choose slightly smaller sucrose clusters with *N* = 1000 because they are computationally less expensive. The 1000-unit particles exhibit a size close to 10 nanometers in diameter and are still in a relevant size regime for single-particle imaging experiments. Their damage mechanisms are similar to the larger particles with 50 nanometers in size even though the underlying nucelar and electron dynamics are a bit faster. In general, the scattering response far away from the resonance is less governed by the electronic structure of the individual atoms but by the complex interplay of the electron and nuclear dynamics, in particular by the electron ejection and delocalization and the interplay with the overall particle scattering response as discussed in detail in Supplementary Note 4.

**Fig. 6:**
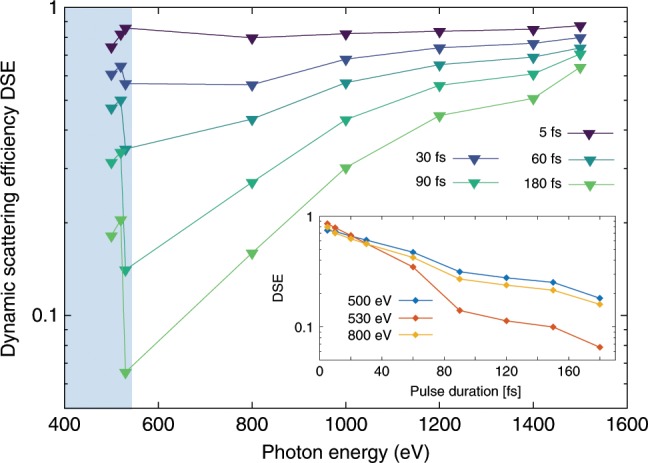
Pulse duration dependence of the diffraction imaging process. Influence of the pulse duration on the beam damage as function of photon energies as well as beam damage for different photon energies as function of pulse length (inset). The calculations are performed for a 1000-unit sucrose cluster exposed to intense XFEL pulses with 25 $\upmu$J/$\upmu$m${}^{2}$. The shaded area indicates the water window.

The calculated DSE for various pulse length as a function of photon energy is shown in Fig. 6. Away from the resonance the calculations indicate less damage for shorter pulses and for pulse durations around the Auger lifetime (5 fs) the scattering response is almost on par with the linear model. Around the oxygen absorption resonance, however, we observe a strong dependence. Here, the pulse duration can modulate the DSE by more than an order of magnitude and the DSE factor approaches one for the shortest pulse, yielding a scattering response comparable to the linear model and undamaged particle, respectively. It is worth noting that at photon energies further below the edge, the overall scattering strength of the oxygen atoms is weaker again and the DSE exhibits less sensitivity for the pulse duration, as the oxygen K electrons become inaccessible for the scattering process. Above the absorption edges, few fs x-ray pulses are required to maintain high elemental contrast from anomalous scattering of ground state atoms before Auger cycling has shifted the resonances out of the bandwidth of the x-ray pulse (Fig. 5). For highly excited systems additional opportunities arise from ultrafast imaging with few femtosecond pulses. Here, the photon energy can be tuned to higher-lying resonances and large contrast from transient states can be obtained^4^ as depicted in Fig. 5.

Based on our data and analysis, concrete recommendations for optimized ultrafast diffractive imaging conditions can be deduced. When few femtosecond pulses with high pulse energy are available, they provide a route for strong scattering signals and enhanced elemental contrast in the resonant regime. In the absence of these pulses, a photon energy sufficiently far away from the absorption resonance should be chosen for reduced x-ray induced damage in the sample. In particular for water window imaging experiments at XFELs, the photon energy needs to be significantly red shifted compared to experiments at synchrotron sources. When using intense XFEL pulses, the linear scattering model cannot be assumed and the total scattering cross section only slightly increases with lower photon energies above the absorption edges. At the absorption edge it exhibits a complicated behavior depending on the pulse length (Fig. 6) and it only recovers clearly below the edge (Fig. 4). Looking at the two investigated data points giving the highest total cross section below and above the oxygen K-edge, i.e., 500 eV and 800 eV, the dependencies on the pulse duration are similar as shown in the insert of Fig. 6. Here it will be important to balance the XFEL performance with the light-induced damage. Choosing a longer pulse length of e.g. 30 femtoseconds will increase the damage only slightly (see insert of Fig. 6). The reason why 30 femtosecond pulses perform well is that they still only can induce limited Auger cycling combined with only small nuclear displacements.

This discussion can also guide feature XFEL mode developments for imaging applications. Here it will be more productive to focus on short pulse modes over total pulse energy output. Recent developments such as the fresh-slice technique have already made large progress in providing few femtosecond pulses with hundreds of micro-Joules output^42,43^. According to Fig. 6 such pulses will outperform the milli-Joule and 180 femtosecond pulses used in the current experiment. The ever increasing number of operation modes for short pulses can further extend the parameter space for imaging experiments^44,45^.

It is noteworthy that for the short pulses comparable to the Auger lifetime, the assumed conditions reach an intensity where stimulated processes and in particular Rabi oscillations can become possible. The rate equation model cannot properly account for such processes but we can offer an additional estimate based on solving the time-dependent Schrödinger equation for a two-level system. We describe the Rabi oscillations between the 1s and 2p in oxygen atoms driven by an intense XFEL pulse with 25 $\upmu$J/$\upmu$m${}^{2}$ and a Gaussian temporal profile with a 5-fs pulse length. We find that the peak Rabi frequency, which scales with the square root of the pulse duration, is 87 times the Auger rate. Under such conditions the measured coherent image is a superposition of the ground and excited state (c.f. Fig. 5) and with the higher probability of interacting with the ground state the DSE in Fig. 6 would move even closer to unity. For longer pulses the stimulated processes are negligible because the participating states are depleted already early in the pulse and the peak intensities are much lower.

We note that imaging biological samples can involve other subshells such as the phosphorus and sulfur atoms at 2145 and 2472 eV, respectively. For higher imaging photon energies in the tender x-ray regime, the resonant ionization conditions presented in this paper for the oxygen edge have to be also considered for the phosphorus and sulfur edges. Specifically, the Auger lifetimes for these edges are much shorter and the Auger cycling will need to be an important factor in the consideration of the imaging conditions.

In conclusion, our atomistic description of the complex x-ray induced phenomena, including correlated electron and nuclear dynamics, with massively parallel codes allows us to describe the main damage mechanisms in large complex samples and give recommendations for optimized experimental parameters at XFELs. Our consistent data set reveals clear deviations from the commonly applied linear scattering models and we show that transient phenomena are detrimental to ultrafast imaging experiments employing intense x-ray pulses with pulse duration >20 fs. As far as 1 keV above the ionization edge, the scattering cross section remains almost flat in contrast to linear scattering experiments and models. Below the ionization edge, transient resonant absorption effectively suppresses the scattering response unless ultrashort pulses resonant with transient electronic configurations are used. In general, single-shot imaging of organic samples in the water window with pulse durations longer than the inner-shell lifetimes need to be performed at much lower energies compared to the linear regime. But near the absorption edge, transient resonances may be exploited for increasing the scattering response and elemental contrast in diffractive imaging experiments.

## Methods

### Single-shot scattering experiment

The imaging experiment of the sucrose (C${}_{12}$H${}_{22}$O${}_{11}$) nanoparticles was carried out at the AMO end station of LCLS in the photon energy range from 500 to 1500 eV^46^. A stream of sucrose clusters was intersected by focused x-ray pulses and their scattering images were recorded by two single photon sensitive, 512 $\times$ 1024 pixel pnCCD detectors^47^ 370 mm behind the interaction region as depicted in Fig. 1. The sucrose clusters were generated by aerosolizing a sugar solution and focusing the resulting dried particles into the interaction region with an aerodynamic lens stack^35,48^. The XFEL delivered pulse energies ranging from 1 to 2 mJ depending on the photon energy. The pulse energies were measured by an upstream gas detector. The uncertainty in pulse energy varied with photon energy between 1% at 530 eV and 25% at 1480 eV. Tuning the XFEL output to the maximum achievable pulse energies also resulted in comparable long pulse durations of 170–180 femtoseconds. For detailed parameters, see Supplementary Note 3.

For the comparison of experimental data to theoretical predictions the knowledge of the peak fluence (photons/cm${}^{2}$) in the interaction point is critical. To characterize the peak fluence, we recorded argon ion-time-of-flight spectra at h$\nu$ = 530 eV from the focal volume within the Rayleigh length^20^ and thus, under similar conditions to the scattering experiment. The resulting data was modeled with Monte Carlo rate equation calculations, containing all photo absorption and inner-shell transition rates as well as focal volume averaging^29^. This approach has been previously shown to accurately reproduce the charge state distribution from atomic targets in intense x-ray pulses^19^. For the current experiment we obtain an average peak fluence of 25 $\upmu$J/$\upmu$m${}^{2}$ at h$\nu$ = 530 eV photon energy, just below the oxygen absorption edge. This is in good agreement with 30 $\upmu$J/$\upmu$m${}^{2}$ obtained by using the measured pulse energy of 1.55 mJ at the gas detectors and typical beam line parameters, namely a transmission of 10% and a focal size of 5 $\upmu$m${}^{2}$^49^. We note that for the higher photon energy data points the beamline transmission was assumed constant even though it can improve at higher energies as the divergence of the x-ray beam becomes smaller and therefore the acceptance of the x-ray transport and focusing optics becomes larger.

### Image analysis and linear scattering model

For fitting the diffraction images we use the linear model previously used for describing single-shot scattering data at LCLS^33–35^. In the linear regime, the particle scattering response depends on its size d or volume V, respectively, the particle refractive index $(1-\Delta n)$, as well as the photon wavelength $\lambda$. Further, the incident fluence on the particle ${I}^{{\rm{o}}}$ as well as the experimental geometry and detector quantum efficiency ${D}_{{\rm{QE,geo}}}$ are required to calculate the number of photons per pixel^35^

1 $${I}_{i}={I}^{{\rm{o}}}{D}_{{\rm{QE}},{\rm{geo}}}{\left(\frac{6\pi V| \Delta n| }{{\lambda }^{2}}\right)}^{2}{\left|\frac{\sin ({s}_{i})-{s}_{i}\cos ({s}_{i})}{{s}_{i}^{3}}\right|}^{2}$$

with ${s}_{i}=\pi d| {{\bf{q}}}_{i}| =\frac{2\pi d}{\lambda }{\mathrm{sin}}(\theta )$. The refractive index for the sugar particles is determined from the form factors of the constituent atoms^50^ and the sucrose mass density of 1581 kg m${}^{-3}$^35^. Using Eq. (1) the incident fluences ${I}^{\text{o}}$ on each particle can be determined from the scattering images and compared to the anticipated incident intensities, ${F}_{0}$, from the beamline data in order to understand the scattering response of samples in the intense x-ray pulse.

### Monte-Carlo/Molecular-Dynamics Method

To understand the scattering response of the sucrose clusters, or more generally organic nanoscale samples, we employed Monte-Carlo/Molecular-Dynamics (MC/MD) calculations^29,36^ to model the full electron and nuclear dynamics in an atomistic manner and calculate the resulting scattering patterns in parallel during the full duration of the x-ray pulse and under realistic beam parameters. The MC/MD approach is similar to the XMDYN method^51^. In more detail, the interaction of the cluster with the incident XFEL pulse is treated quantum mechanically with a Monte Carlo method by tracking explicitly the time-dependent quantum transition probability between different electronic configurations. The total transition rate, $\Gamma$, between different electronic configurations $I$ and $J$ is given by

2 $${\Gamma }_{I,J}={\Gamma }_{I,J}^{P}+{\Gamma }_{I,J}^{A}+{\Gamma }_{I,J}^{F}+{\Gamma }_{I,J}^{RE}+{\Gamma }_{I,J}^{EI}+{\Gamma }_{I,J}^{RC}.$$

Starting from the ground state of the neutral atom, we include the contribution from photoionization ${\Gamma }_{I,J}^{P}$, Auger decay ${\Gamma }_{I,J}^{A}$, fluorescence ${\Gamma }_{I,J}^{F}$, resonant excitation ${\Gamma }_{I,J}^{RE}$, electron-impact ionization ${\Gamma }_{I,J}^{EI}$, and recombination ${\Gamma }_{I,J}^{RC}$. Further, the model includes resonant excitation processes, which have been demonstrated to play a crucial role in atomic ionization in intense x-ray pulses close to absorption edges^17,19,38,52^. Additionally, a molecular dynamics (MD) algorithm is used to propagate all particle trajectories (atoms/ions/electrons) forward in time in 10 attosecond steps.

The importance of understanding transient dynamics is that the incoming photons arriving at different times will scatter off the instantaneously populated transient states. Similar to the treatment presented in refs. ^29,36^, the observed scattering response is characterized as a sum of the instantaneous scattering patterns weighted by the pulse intensity, ${j}_{X}(\tau ,t)$, with FWHM duration $\tau$ and convolved with a Gaussian bandwidth profile, $g(\omega ,{\omega }_{x})$, with a central photon energy of ${\omega }_{x}$, such that

3 $$\frac{d{\sigma }_{{\rm{dam}}}}{d\Omega }({\boldsymbol{q}})=\frac{d{\sigma }_{{\rm{th}}}}{d\Omega }\frac{1}{{\mathscr{F}}}\int_{0}^{+\infty} d\omega \int_{-\infty}^{+\infty} dtg(\omega ,{\omega }_{x}){j}_{X}(\tau ,t)[| {F}_{c}({\boldsymbol{q}},t){| }^{2}+{N}_{e}(t)],$$

where ${N}_{e}(t)$ is the number of delocalized electrons, $d{\sigma }_{{\rm{th}}}/d\Omega$ is the Thomson scattering cross section.

4 $${\mathscr{F}}=\int_{0}^{+\infty} d\omega \int_{-\infty}^{+\infty} dt{j}_{X}(\tau ,t)g(\omega ,{\omega }_{x})$$

is the fluence of an XFEL pulse, and ${\int }_{0}^{+\infty }d\omega (\omega ,{\omega }_{x})=1$. Here ${F}_{c}({\boldsymbol{q}},t)$ is the time-dependent form factor of the target cluster and is given by

5 $${F}_{c}({\boldsymbol{q}},t)=\sum _{j=1}^{{N}_{a}}{f}_{j}({\boldsymbol{q}},{C}_{j}(t)){e}^{i{\boldsymbol{q}}\cdot {{\boldsymbol{R}}}_{j}(t)}\ ,$$

where ${C}_{j}(t)$ and ${{\boldsymbol{R}}}_{j}(t)$ are, respectively, the electronic configuration and position of the $j$-th atom/ion at time $t$, ${N}_{a}$ is the number of atoms/ions, and ${f}_{j}({\boldsymbol{q}},{C}_{j}(t))$ is the complex atomic form factor of the $j$-th atom/ion

6 $${f}_{j}({\boldsymbol{q}})={f}_{0,j}({\boldsymbol{q}},{C}_{j}(t))+{f}_{j}^{{\prime} }(\omega )+i{f}_{j}^{{\prime\prime} }(\omega )$$

with

7 $${f}_{0,j}({\boldsymbol{q}},{C}_{j}(t))=\int_{-\infty}^{+\infty} d{\boldsymbol{r}}\rho ({\boldsymbol{r}},{C}_{j}(t)){e}^{i{\boldsymbol{q}}\cdot {\boldsymbol{r}}}$$

which is just the Fourier transform of electron density, $\rho ({\boldsymbol{r}},{C}_{j}(t))$, and ${\boldsymbol{q}}$ is the momentum transfer vector. Here, $f^{\prime}$ and $f^{\prime\prime}$ are the real and imaginary parts of the anomalous (resonant) scattering terms, and they are related to Kramers-Kronig relation^50,53^. These terms depend on the photon energy and give rise to rapid modulation of the form factor near absorption edges and resonances. In particular, for each electronic configuration, $f^{\prime\prime}$ is computed using the optical theorem^54^,

8 $$f^{\prime\prime} (\omega )=\sum _{v}\frac{\omega }{4\pi \alpha }{\sigma }_{v}^{({\rm{PI}})}+\sum _{u,v}\frac{\pi }{2}{\omega }_{vu}{\,}{f}_{vu}^{({\rm{osc}})}\frac{1}{\pi }\frac{{\gamma }_{vu}/2}{{(\omega -{\omega }_{vu})}^{2}+{\gamma }_{vu}^{2}/4}$$

where ${\sigma }_{v}^{(\,\text{PI}\,)}$ is the photoionization cross section of the occupied $u$-subshell, ${\omega }_{vu}$ is the transition energy of electron in the occupied $v$-subshell to the $u$-subshell with a natural linewidth of ${\gamma }_{vu}$, and ${f}_{vu}^{({\rm{osc}})}$ is the oscillator strength of this dipole transition. We note that our approach does not account for vibrational broadening.

The total scattering cross section is then computed as

9 $${\sigma }_{{\rm{dam}}}=\int d{\boldsymbol{\Omega }}\frac{d{\sigma }_{{\rm{dam}}}}{d\Omega }({\boldsymbol{q}})$$

Compton scattering was not included as its total cross section is small for the energy range considered.

We note that in Eq. (3), the scattering of the delocalized electrons is treated as an incoherent scattering process^55,56^. The total cross sections computed from this treatment are similar to those computed with by including scattering amplitude of these electrons explicitly^57^ (see Supplementary Note 5). The small difference between the treatment of^57^ and our treatment is a result of a large fraction of delocalized electrons escape the cluster volume, such that the average distance between these electrons is much larger than the x-ray wavelength. As a result, the scattering amplitudes of these electrons do not add in phase, and they contribute linearly to the scattering signal.

### Dynamic scattering efficiency

We introduce the Dynamic scattering efficiency (DSE) for comparing the calculated cross sections with the experimental measurements. It is a dimensionless quantity that quantifies the x-ray induced suppression/enhancement of the particle scattering response. It describes the reduction/enhancement in the total number of scattered photons ${N}_{{\rm{scatt}}}$ over the linear models with the undamaged cross section ${\sigma }_{{\rm{nodam}}}$ and the absolute fluence ${F}_{0}$ impinging on the particle through

10 $${N}_{{\rm{scatt}}}={F}_{0}\ {\sigma }_{{\rm{dam}}}={F}_{0}\ DSE\ {\sigma }_{{\rm{nodam}}}.$$

For the experimental data (see Fig. 3), DSE is the ratio of the fitted fluence, ${I}^{{\rm{o}}}$, over the peak fluence, ${F}_{0}$, and for the theoretical results DSE is the ratio of the damaged to the undamaged cross sections ${\sigma }_{{\rm{dam}}}/{\sigma }_{{\rm{nodam}}}$. The advantage of using and discussing the DSE is that it allows direct comparison of the experimental data to the calculated cross sections.

## Supplementary information

Supplementary Information

## Data Availability

Source data have been deposited with the Coherent X-ray Imaging Databank (CXIDB) with the accession identifier CXIDB ID 119 [10.11577/1574692]. Data deposition with CXIDB includes: Preprocessed LCLS data files (/preprocessed); Fluence analysis (/sizing). Other data are available from the corresponding authors upon reasonable request.

## Code availability

Data processing scripts and sucrose fluence analysis examples are available from this public code repository: http://github.com/fxihub/sucrose-cluster-analysis. The code for the MD/MC calculations is described in detail in ref. 29. It will be available upon reasonable request.
