# Supplementary material for: The role of transient resonances for ultra-fast imaging of single sucrose nanoclusters
Source: Nat Commun. 2020 Jan 9;11:167. doi: 10.1038/s41467-019-13905-9 (PMC6952381; doi:10.1038/s41467-019-13905-9)
Supplement: Supplementary file 1 — Supplementary Information [file 41467_2019_13905_MOESM1_ESM.pdf]

Supplementary Information

The role of transient resonances for ultra-fast imaging of single sucrose nanoclusters

Ho *et al.*

SUPPLEMENTARY NOTE 1: SIZE DISTRIBUTIONS OF SUCROSE CLUSTERS

The size of the individual sucrose cluster can be in principle determined in linear scattering theory using eq. (1) from the main text. In the vicinity of an absorption resonance, however, the refractive index is only poorly described by the Henke tables [1] for which bound-bound transitions are not included. This can lead to a skewing of the determined sizes. Additionally there will be different degrees of damage depending on the photon energies, contributing to uncertainties in the particle sizes determined from the linear scattering model fits.

In the following we show that the main conclusions are independent from the details in the sample size distribution. In Supplementary Fig. 1 we plot the size distribution of the sucrose clusters recovered from the scattering pattern for each photon energy. The average size varies across different photon energies, in particular at the resonant energy. Specifically, the mean cluster diameters are 37.1 nm, 48.1 nm, 49.2 nm, 48.6 nm, 53.8 nm at 530 eV, 800 eV, 1000 eV, 1140 eV and 1483 eV respectively.

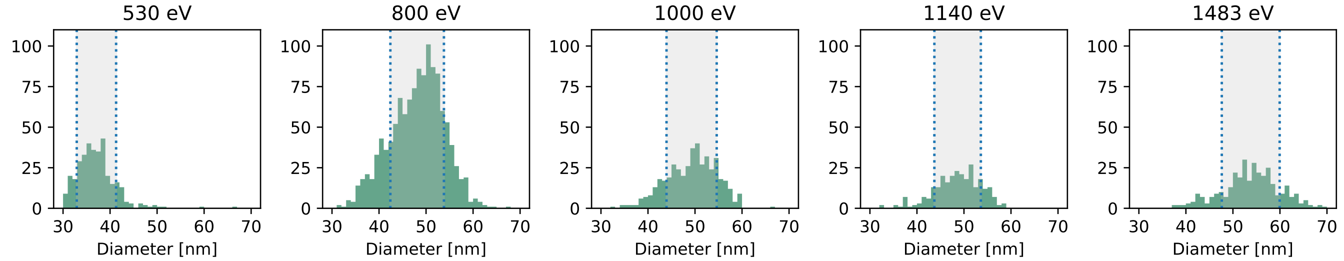

Supplementary Fig. 1. Distributions of sucrose cluster sizes for 5 photon energies. The shaded area shows ± one standard deviation from the mean cluster size determined from the measured scattering patterns.

Photon Energy (eV) (eV)	d_{all} (nm) (nm)	$d_{\mathrm{all},5\%}$ (nm) (nm)	$d_{\mathrm{sd},5\%}$ (nm)% (nm)
530	37.1 ± 4.2	37.5 ± 5.3	36.9 ± 1.8
800	48.1 ± 5.7	47.6 ± 5.5	47.9 ± 3.1
1000	49.2 ± 5.3	48.3 ± 4.7	48.8 ± 3.2
1140	48.6 ± 5.0	48.0 ± 5.1	48.8 ± 2.3
1484	53.8 ± 6.2	49.0 ± 6.8	53.8 ± 3.5

Supplementary Table 1. Cluster size statistics. Mean cluster diameter and the standard deviation as function of photon energy for all clusters (30-70) nm (d_{all}), all clusters with top 5% most intense shots ($d_{\mathrm{all},5\%}$) and clusters with size falls within one standard deviation and top 5% most intense shots ($d_{\mathrm{sd},5\%}$).

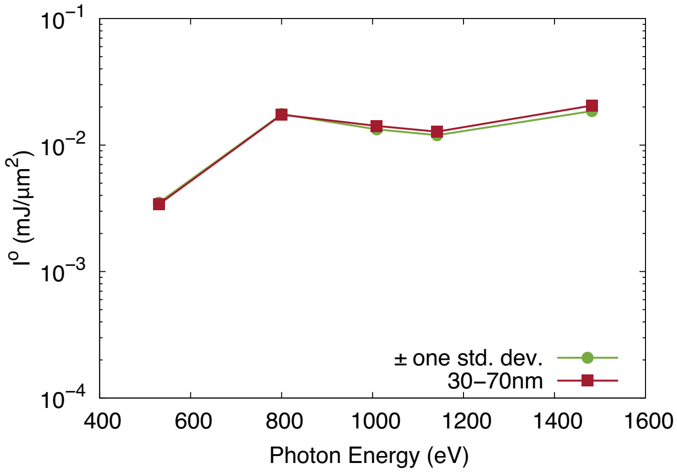

Supplementary Fig. 2. Maximum fluence as a function of photon energy. Fitted incident fluence I^o for the 5% most intense hits at each photon energy for clusters with sizes 30-70 nm and within one standard deviation of the mean.

Figure 3 in the main text presents the I^o obtained for top 5 % most intense shots of clusters with sizes falls within the ± one standard deviation from the mean (the shaded region in 1). Similar photon energy dependence of I^o can be obtained for top 5 % most intense shots images from all the clusters with sizes 30-70 nm. For each photon energy, these subsets of clusters have similar mean diameter and standard deviation, as shown in Table 1. This means that the top 5% intense shots used in the Figure 3 of the main text provide a good statistical representation of all the sucrose clusters used in the experiment, and the photon energy dependence of I^o is not statistically biased.

SUPPLEMENTARY NOTE 2: ERROR ESTIMATION FOR SUCROSE DIFFRACTION FITTING PROCEDURE

For a given sucrose diffraction pattern non-spherical features as well as background and noise characteristics can affect the sphere model fitting procedure (described in Methods) used to determine the particle size and fluence of a single shot. To estimate the confidence interval of the energy-dependent fluence values presented in Fig. 3. of the main text, we selected the lowest of the 5% most intense events. This selection represents the worst case among the relevant data points shown in Fig. 3. and 4 in the main text.

Figure S1. shows the selected diffraction patterns (top row) compared to the Poisson-sampled sphere diffraction model (second row). For a given pixel i, the measured photon count is depicted n_i and the fitted photon count is I_i using the sphere model described in eq.1 (Methods). For this error analysis, we consider azimuthally averaged photon counts $n(q)$ and $I(q)$ where q is the radial spatial frequency in units of nm^{-1}. The averages $n(q)$ and $I(q)$ and are shown in the third row of Figure S3. We assume a Gaussian noise model for the averaged photon counts and write the likelihood $\mathcal{L}$ as the combined probability of measuring $n(q)$ given $I(q)$

$$\mathcal{L}(n|I,\sigma) = \prod_q \frac{1}{\sqrt{2\pi\sigma^2}} \exp\left[-\frac{(n(q)-I(q))^2}{2\sigma^2}\right].$$

We assume σ to be the same for all frequencies q, ignore a global scaling factor and rewrite the likelihood as

$$\mathcal{L}(n|I) \propto \exp\left[-\sum_q (n(q)-I(q))^2\right].$$

In a brute-force approach, we can create a map $\mathcal{L}(n|I(I^0+\Delta I^0, d+\Delta d))$ where I^0, d are the fitted values for fluence and particle, and $\Delta I^0, \Delta d$ given deviations from those values. The full likelihood map is normalised such that $\mathcal{L}(n|I(I^0,d)) = 1$ and is shown in the bottom row of Figure S3. For the estimation of the confidence intervals, we determined the contour line in the likelihood map which contains 95% of the integrated map (red-dashed line). The confidence intervals are then defined as the lower and upper bound of that contour line for $\Delta I^0/I^0$ and Δd respectively.

The following table, summarises the obtained error estimates for the sucrose diffraction fitting procedure:

Energy (eV)	Fluence I^0 (μJ/μm^2)	Lower bound at 95% (μJ/μm^2)	Upper bound at 95% (μJ/μm^2)	Lower bound at 68% (μJ/μm^2)	Upper bound at 68% (μJ/μm^2)
529.8	1.77	1.49 (-16%)	2.13 (+20%)	1.60 (-10%)	1.97 (+11%)
797.9	10.82	10.61 (-2%)	11.05 (+2%)	10.72 (-1%)	10.94 (+1%)
1009.6	7.81	7.50 (-4%)	8.05 (+3%)	7.65 (-2%)	7.97 (+2%)
1140.8	8.39	7.30 (-13%)	9.57 (+14%)	7.72 (-8 %)	9.15 (+9%)
1483.9	10.95	9.41 (-14%)	12.48 (+14%)	10.07 (-8 %)	11.82 (+8%)

Energy (eV)	Diameter d (nm)	Lower bound at 95% (nm)	Upper bound at 95% (nm)	Lower bound at 68% (nm)	Upper bound at 68% (nm)
529.8	38.9	37.1	40.7	37.8	40.0
797.9	45.9	45.5	46.3	45.7	46.1
1009.6	50.7	49.7	51.6	50.1	51.2
1140.8	51.0	49.6	52.3	50.2	51.8
1483.9	56.1	55.0	57.0	55.2	56.6

Supplementary Table 2. Fluence and size error bounds. The top part of the table shows the error bounds for the fluence estimate as a function of incident photon energy. The lower section of the table shows the error bounds for the size estimate of the sucrose nanoclusters.

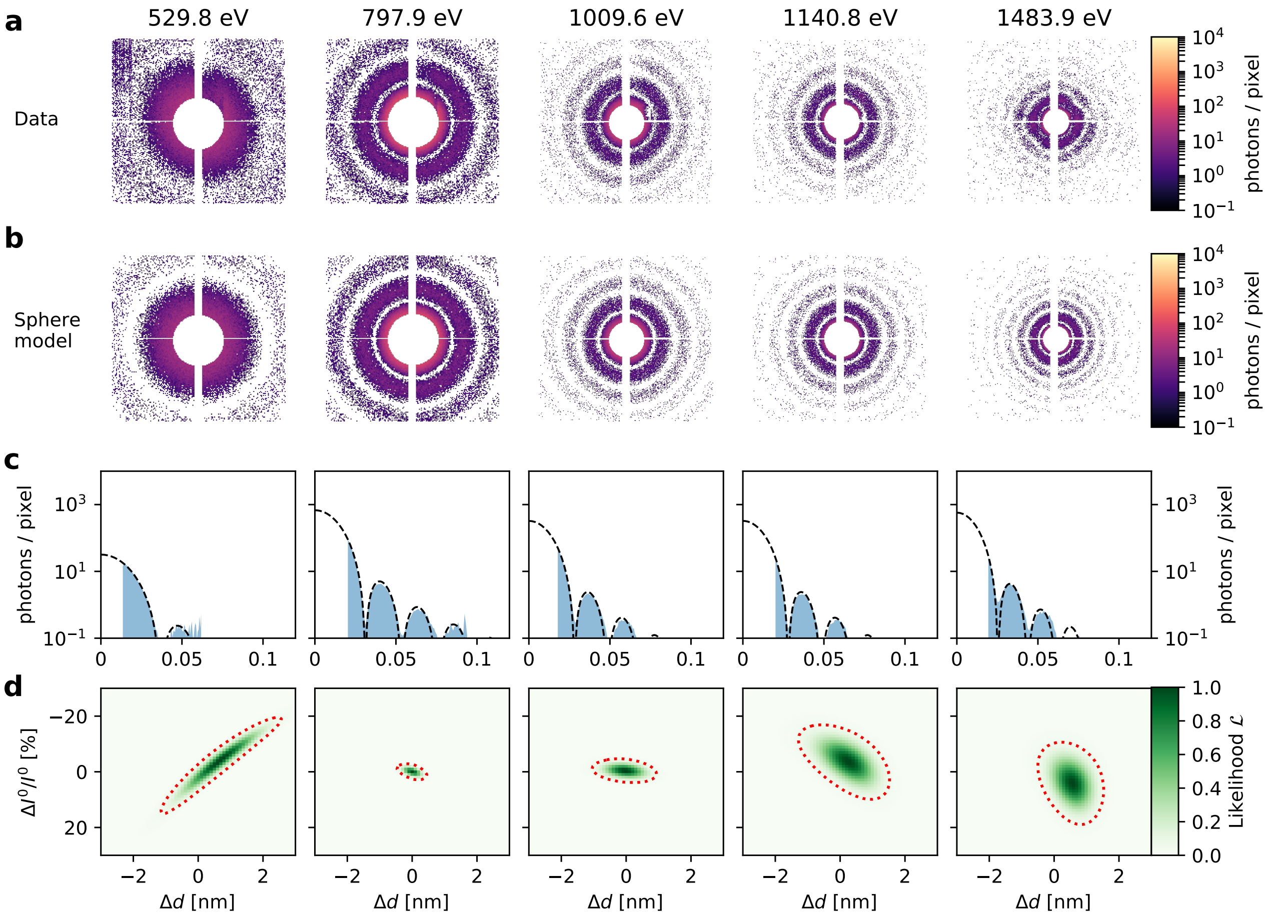

Supplementary Fig. 3. Error analysis of the sucrose diffraction fitting procedure. For each photon energy presented in Fig. 3 of the main text, the lowest of the 5% most intense events is selected. The corresponding diffraction patterns are shown in (a). The Poisson-sampled sphere diffraction model at the estimated (fitted) size and intensity parameters is shown in (b). Radial averages of the diffraction data (blue shade) and sphere model (black-dashed line) are depicted in (c). The normalised likelihood of observing the measured photon counts given the modelled intensities is plotted in (d) assuming a Gaussian noise model mapped for different size and intensity parameters. The red-dashed line indicates an area corresponding to 95 % of the integrated likelihood map.

SUPPLEMENTARY NOTE 3: UNCERTAINTIES IN BEAM ENERGIES AND PULSE DURATIONS

Supplementary Table 3 lists the mean and deviation of pulse duration obtained from the accelerator parameters and pulse energy obtained from the gas monitor detector for all the images shown in Fig. 2 of the main text. For each photon energy, the variation in pulse energy is at most 25%. To obtain the peak fluence, we normalized the beamline data to Ar time-of-flight data taken under similar conditions as the scattering experiment and modeled it with Monte Carlo rate equation calculations as described in the main text. This approach has been previously shown to accurately reproduce the charge state distribution from atomic targets in intense x-ray pulses [2].

Photon Energy (eV)	Pulse Duration (fs)	Pulse Energy (mJ)
530	175.31 ± 1.62	1.55 ± 0.02
800	172.37 ± 5.54	1.89 ± 0.33
1000	174.57 ± 1.95	1.71 ± 0.36
1140	175.69 ± 0.93	1.01 ± 0.27
1184	177.93 ± 0.98	1.52± 0.38

Supplementary Table 3. Mean and standard deviation of pulse duration and the pulse energy obtained from the gas monitor detector.

SUPPLEMENTARY NOTE 4: CALCULATED ELECTRON DENSITY DISTRIBUTION OF A 1000 UNIT SUCROSE PARTICLE AWAY FROM RESONANCES

For understanding the scattering response of the sucrose particles it is important to discuss the electron distribution of the excited particle in detail. In a homogeneous particle and under a hydrodynamic expansion the ion and electron distribution inside the particle are, at least theoretically, more or less equivalent [3] whereas in inhomogenous particles and for higher photon energies a rather complex interplay can be anticipated. Early experiments on methane clusters at the FLASH free-electron laser with XUV radiation have already shown that the protons can efficiently remove charge from the nanoplasma within the femtosecond pulse leading to a distinct final kinetic energy difference in protonated and deuterated clusters [4].

To investigate electron dynamics and losses, we examined the time-dependent electron density of a 1000-unit sucrose cluster exposed to a 800-eV, 180-fs XFEL pulse with 25 μJ/μm^2. The results are shown in Supplementary Fig. 4. We found that the cluster undergoes significant (electronic and structural) changes during the interaction with the x-ray pulse. In our case of an intense x-ray pulse, the single ionization (carbon and oxygen) is saturated - meaning that there is unit probability of producing a singly charged ion and significant probability to produce higher charge states. Also, the x-ray pulse duration is much longer than the inner-shell lifetime of the ionized carbon and oxygen atoms. As a consequence, Auger decay and further sequences of ionization and Auger decay can take place with high probability to enable production of very high charge states atoms during the pulse [5].

During the interaction with the pulse the number of electrons remaining within the initial particle radius is greatly and rapidly reduced (Supplementary Fig. 4 a). Near the end of the pulse (t = 125 fs), the electron density of the cluster in the original cluster volume is reduced by close to a factor of ten. In more detail (Supplementary Fig. 4 b-h), the ionized electrons escape far away from the original volume, occupying a volume multiple times the initial cluster volume. The structure expands early on and long before the peak intensity of the x-ray pulse is reached. The atoms are highly ionized such that the density of bound electrons is reduced. The fraction of ionized electrons that remain in the original cluster volume is small in comparison to the fraction of escaped electrons.

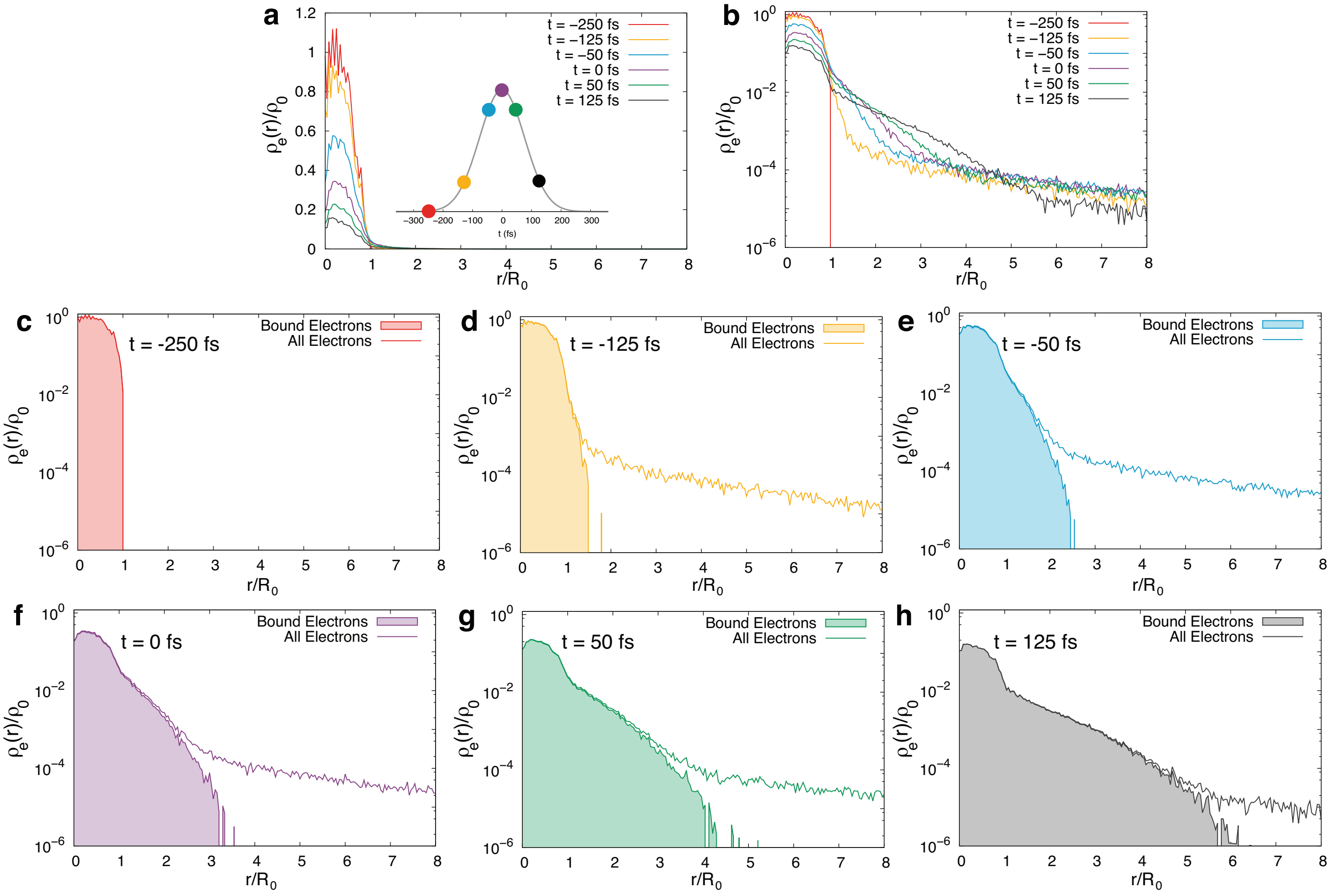

Supplementary Fig. 4. Evolution of the electron density of a sucrose cluster during an XFEL pulse. (a) Electron density $\rho_e(r)$ of sucrose cluster at different times during an 180-fs pulse. The electron density includes all electrons (bound and unbounded). The inset shows the chosen time points in the XFEL pulse. Here ρ_0 and R_0 are the average electron density and cluster size of undamaged cluster. Plot (b) is same (a), but the electron density is plotted in log scale. The electron density of all electrons (solid line) and only bound electrons (filled curves) at (c) t = -250 fs, (d) t = -125 fs, (e) t = -50 fs, (f) t = 0 fs, (g) t = 50 fs and (h) t = 125 fs. The calculations are performed for a 1000-unit sucrose cluster exposed to intense XFEL pulses with 25 μJ/μm^2.

SUPPLEMENTARY NOTE 5: SCATTERING CALCULATION: COHERENT VS INCOHERENT CONTRIBUTION OF FREE ELECTRONS

In our model of the scattering response we treat the delocalized electrons with a linear or in other words incoherent scattering response as shown in eq. (3) in the main text. This is justified based on their rapid and large delocalization compared to the initial cluster volume under irradiation with intense x-ray pulses discussed in the previous section and shown in Supplementary Fig. 4. We note that this is a different approach to the one chosen by Peltz at al. [3] who work in a hydrodynamic model and who have a near perfect congruence of the electron and ion density profiles. Peltz et al. calculate the scattering amplitudes of the delocalized electrons explicitly. In the following paragraphs we will first argue which terms we can neglect based on the rapid electron delocalization, occupying multiple times the volume of the initial cluster, for scattering into larger q values. Then we show that both approaches yield essentially the same results for particles in intense x-ray pulses with large electron delocalization. We note that our approach has the advantage that we reduce from a quadratic to a linear scaling in computational cost and therefore large systems become accessible for modeling.

We start with the approach by Peltz et al. in which the scattering amplitudes of the delocalized electrons are described as

$$\frac{d\sigma_{\mathrm{dam}}}{d\Omega}(\boldsymbol{q}) = \frac{d\sigma_{\mathrm{th}}}{d\Omega}\frac{1}{\mathscr{F}}\int_{-\infty}^{+\infty} dt j_X(\tau,t)|F_{\mathrm{quad}}(\boldsymbol{q},t)|^2, \tag{1}$$

where

$$F_{\mathrm{quad}}(\boldsymbol{q},t) = F_c(\boldsymbol{q},t) + \sum_{j=1}^{N_e(t)} e^{i\boldsymbol{q}\cdot\boldsymbol{r}_j(t)}\,, \tag{2}$$

where $F_c(\boldsymbol{q},t)$ is the time-dependent composite form factor of the atoms/ions in target cluster

$$F_c(\boldsymbol{q},t) = \sum_{j=1}^{N_a} f_j(\boldsymbol{q},C_j(t))e^{i\boldsymbol{q}\cdot\boldsymbol{R}_j(t)}\,, \tag{3}$$

where $C_j(t)$ and $\boldsymbol{R}_j(t)$ are, respectively, the electronic configuration and position of the j-th atom/ion at time t, N_a is the number of atoms/ions, $f_j(\boldsymbol{q},C_j(t))$ is the complex atomic form factor of the j-th atom/ion, $\boldsymbol{r}_j(t)$ position of the j-th electron at time t and $N_e(t)$ is the number of electrons.

From eq. (2) and (3), we get

$$|F_{\mathrm{quad}}(\boldsymbol{q},t)|^2 = |F_c(\boldsymbol{q},t)|^2 + N_e(t) + \sum_{j,k=1;j\neq k}^{N_e(t)} e^{i\boldsymbol{q}\cdot(\boldsymbol{r}_k(t)-\boldsymbol{r}_j(t))} + \left(\sum_{j=1}^{N_e(t)} e^{-i\boldsymbol{q}\cdot\boldsymbol{r}_j(t)}F_c(\boldsymbol{q},t) + c.c.\right). \tag{4}$$

For our sucrose cluster, a large fraction of electrons escape the cluster volume during the pulse and the distances between escaped electrons and ions/atoms are much larger than the size of the undamaged cluster, see (Supplementary Fig. 4). Under these conditions, the third and fourth term are negligible and the quadratic coherent treatment of free-electron scattering is equivalent to treating it as an incoherent scattering process.

As a result of this large spatial extension of the free-electron cloud away from the cluster, we found that the free-electron scattering can be treated as an incoherent scattering process as they essentially loose their phase correlation. This reduction of electron density leads to drastic changes in the differential cross section during the pulse (see Supplementary Fig. 5 (a)), and a substantial loss in scattering power in comparison to that of the undamaged sample (see Supplementary Fig. 5 (c)). Supplementary Fig. 5 (c) shows that the differential cross section computed by this treatment agrees well with the one calculated by treating the free-electron scattering as a coherent process [see Peltz et al., Phys. Rev. Lett. **113**, 133401 (2014)], in particular for the large angles observed in the experiments.

Further, we made a comparison between the treatment of coherent and incoherent free-electron scattering at photon energies of 530 eV, 1000 eV and 1500 eV for a 1000-unit sucrose cluster exposed to an 180-fs XFEL pulse with 25 μJ/μm^2. Supplementary Fig. 6 show that the differential cross sections from these two methods are very similar, with the largest difference at very small q values (<0.01 Å^{-1}). To calculate the DSE, we compute the ratio of σ_{dam} and σ_{nodam}. Note that σ_{dam} is computed from eq (9) of the main text, in which the differential cross section is weighted by the solid angle, such that the contribution in the forward direction is suppressed (see Supplementary Fig. 5 (d)). As a result, the DSE values from the coherent treatment changes only by about 5% at 530 eV (less than 1% at 1500 eV) and the result does not affect our finding that resonant excitation and a 180-fs pulse suppress the scattering signal by an order of magnitude.

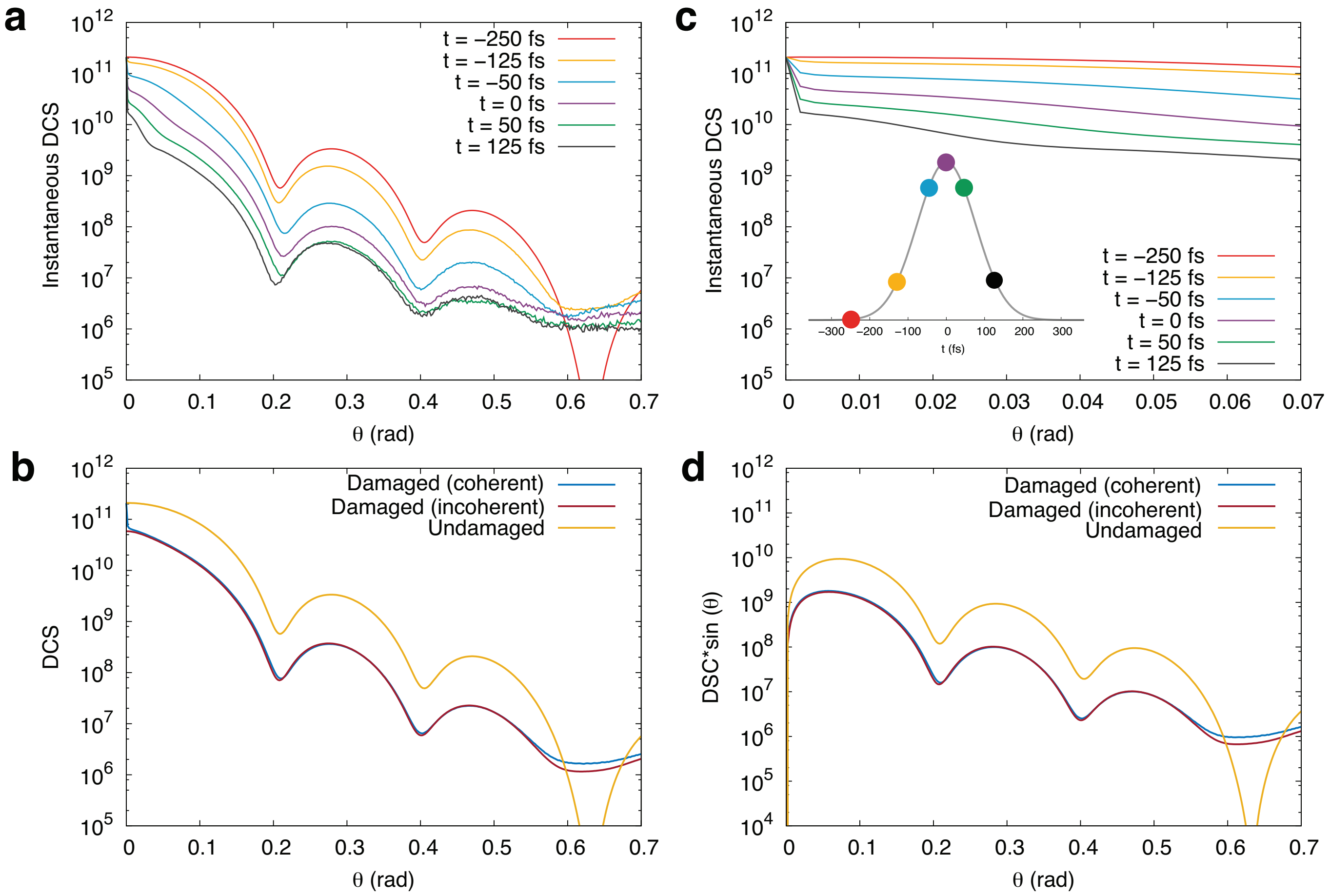

Supplementary Fig. 5. Change in the differential cross section of sucrose nanoclusters throughout the XFEL pulse. (a) Instantaneous differential cross sections (DCS) of a 1000-unit sucrose cluster at different times during a 180-fs XFEL pulse with a photon energy of 800 eV and a fluence of 25 μJ/μm^2. DCS are calculated by treating free-electron scattering as a coherent process. Plot (b) is same as (a), but it is plotted for a smaller range of scattering angles. The inset shows temporal profile of the x-ray flux and the chosen time points in the XFEL pulse. (c) Time integrated differential cross sections of dynamically changing sucrose cluster obtained using the treatment of coherent (blue line) and incoherent (red line) free electron scattering. For comparison, DCS assuming no damage is shown in yellow line. (d) DCS weighted by $\sin(\theta)$ obtained using the treatment of coherent (blue line) free-electron scattering agree with that calculated using the treatment of incoherent (red line) free-electron scattering.

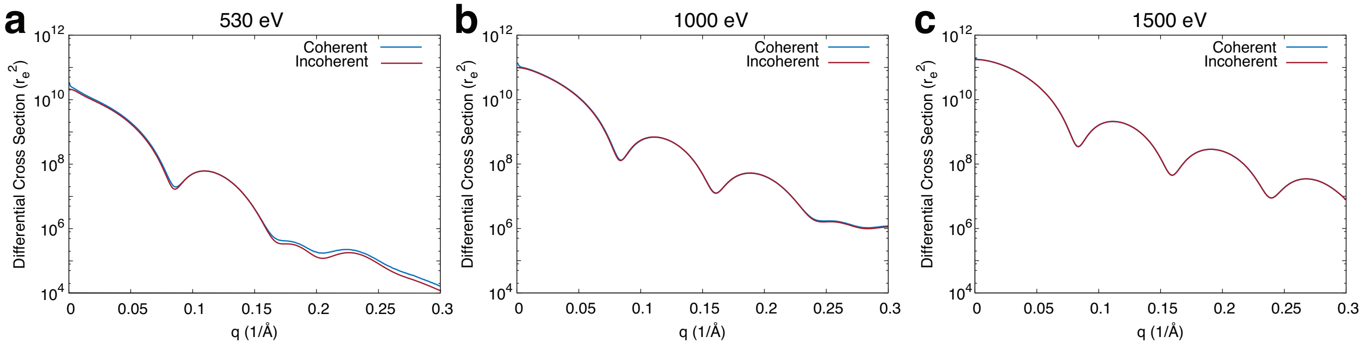

Supplementary Fig. 6. Comparison between coherent and incoherent calculations of the differential cross sections. Differential scattering cross sections obtained using coherent and incoherent contribution of free electrons for (a) 530 eV (b) 1000 eV and (c) 1500 eV. The calculations are performed for a 1000-unit sucrose cluster exposed to intense XFEL pulses with 25 μJ/μm^2.

SUPPLEMENTARY NOTE 6: ELECTRONIC AND IONIC DAMAGE AT THE RESONANCE

To monitor the structural dynamics of a 50-nm sucrose cluster, we plot the ratio of the rms radius, $S(t)$, of the XFEL-excited cluster and of that the undistorted structure, S_0 as a function of time for 180-fs, 530-eV pulse with 25 μJ/μm^2 in Supplementary Fig. 7. Here, a very dense nanoplasma is formed due to the resonant excitation conditions[6]. At the peak of the pulse the cluster has already expanded by a factor of 20% on average. We note that the distortion of the structure is not spatially uniform. The average distance between atoms/ions in the shell layers increases much faster than that in the cluster core, consistent with previous theoretical descriptions [7] and experimental observations [8]. The dynamic expansion of the particle has consequences for the scattering process. First, the rapid expansion of the particle leads to a re-flattening of the Coulomb potentials and more electrons can leave the particle [9] than a static model would suggest [10]. Specifically, for the sucrose particles at the modeled conditions and 530 eV, more than 30% of the electrons escape at the peak of the pulse. Second, the shrinking solid-density particle core and surface softening will also reduce the scattered intensity as previously described from superheated cluster in intense laser fields [8].

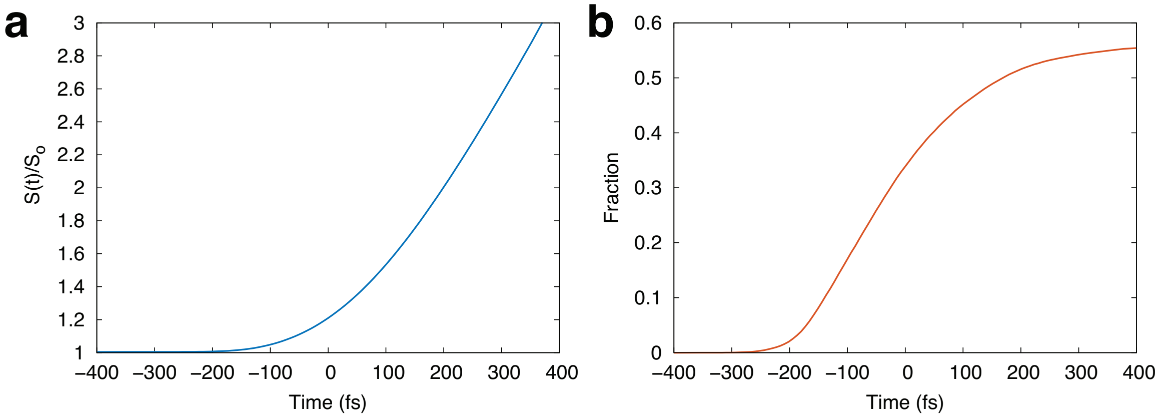

Supplementary Fig. 7. Expansion of the electron cloud. (a) Ratio of the rms radius, $S(t)$, to the undistorted structure, S_0 as a function of time. (b) Fraction of the electron leaving the undistorted cluster volume.

SUPPLEMENTARY REFERENCES

[1] Henke, B. L. & DuMond, J. W. M. Submicroscopic structure determination by long wavelength xray diffraction. *Journal of Applied Physics* **26**, 903–917 (1955).

[2] Ho, P. J., Bostedt, C., Schorb, S. & Young, L. Theoretical tracking of resonance-enhanced multiple ionization pathways in x-ray free-electron laser pulses. *Phys. Rev. Lett.* **113**, 253001 (2014).

[3] Peltz, C., Varin, C., Brabec, T. & Fennel, T. Time-resolved x-ray imaging of anisotropic nanoplasma expansion. *Phys. Rev. Lett.* **113**, 133401 (2014).

[4] Iwan, B. *et al.* Explosion, ion acceleration, and molecular fragmentation of methane clusters in the pulsed beam of a free-electron laser. *Phys. Rev. A* **86**, 033201 (2012).

[5] Young, L. *et al.* Femtosecond electronic response of atoms to ultra-intense x-rays. *Nature* **466**, 56–61 (2010).

[6] Bostedt, C. *et al.* Fast electrons from multi-electron dynamics in xenon clusters induced by inner-shell ionization. *New Journal of Physics* **12**, 083004 (2010).

[7] Hau-Riege, S. P., London, R. A. & Szoke, A. Dynamics of biological molecules irradiated by short x-ray pulses. *Phys. Rev. E* **69**, 051906 (2004).

[8] Gorkhover, T. *et al.* Femtosecond and nanometre visualization of structural dynamics in superheated nanoparticles. *Nat. Photon.* **10**, 93–97 (2016).

[9] Fennel, T. *et al.* Laser-driven nonlinear cluster dynamics. *Rev. Mod. Phys.* **82**, 1793–1842 (2010).

[10] Bostedt, C. *et al.* Multistep ionization of argon clusters in intense femtosecond extreme ultraviolet pulses. *Phys. Rev. Lett.* **100**, 133401 (2008).
